# Leiomyoma of the Nipple: A Rare Entity at Rare Site

**DOI:** 10.7759/cureus.62220

**Published:** 2024-06-12

**Authors:** Nimisha Dhankar, Reena Tomar, Sreoshi Paul, Nita Khurana, Sushanto Neogi

**Affiliations:** 1 Pathology, Maulana Azad Medical College, New Delhi, IND; 2 Surgery, Maulana Azad Medical College, New Delhi, IND

**Keywords:** rare site, benign, immunohistochemistry, breast, nipple leiomyoma

## Abstract

Cutaneous leiomyomas are benign and rare smooth muscle tumors. Genital leiomyoma includes leiomyomas located in the nipple, scrotum, areola or vulva. Nipple leiomyomas are the least common genital leiomyomas and are commonly seen to occur in middle-aged women. Here, we present a case of a 40-year-old female complaining of a growth over the right nipple for six months. On local examination, it was a 1cm x 1cm growth on the lateral aspect of the nipple. Finally, a treatment plan of complete excision was done. Grossly, it was a well-circumscribed skin-covered soft tissue bit with a skin flap measuring 0.6cm x 0.6cm and soft tissue measuring 0.6cm x 0.5cm x 0.2cm. Histopathological examination revealed a skin-covered section with dermis showing a well-circumscribed unencapsulated lesion that showed intersecting fascicles of spindle cells with no atypia or mitoses noted. Microscopically, the growth had tumor-free resection margins. Immunohistochemical confirmation with S100, smooth muscle actin (SMA) and caldesmon was done. Diagnosis of nipple leiomyoma was given with strong SMA positivity. Nipple leiomyoma is a rare, benign lesion and needs to be correctly diagnosed microscopically. Biopsy and immunohistochemistry is a confirmatory investigation that can lead to timely management of the patient.

## Introduction

Leiomyoma is a rare, benign, smooth muscle cell tumor that can arise virtually anywhere in the body where smooth muscle tissue is present. Cutaneous leiomyomas are rare and can be broadly divided into three groups based on the cell of origin: pilar leiomyomas, angioleiomyomas, and genital-type leiomyomas (scrotal, vulval and nipple) [1]. Genital leiomyomas are very uncommon and arise from the dartos muscle of the scrotum/labia majora and the erectile smooth muscle of the nipple. Out of these, nipple leiomyomas have the least prevalence [1]. These leiomyomas have a benign course, however recurrence can be seen. This case report discusses the clinical, histological, and immunohistochemical characteristics and management of a unilateral nipple leiomyoma in an adult female.

## Case presentation

We report a case of nipple leiomyoma in a 40-year-old lady who presented to surgery OPD with a lump over the right nipple for the last six months. The growth was firm, gradually increasing in size, and was painful. There was no associated nipple discharge or ulceration. She had no other breast or axillary lump and the skin over the nipple and breast was unremarkable. There was no history of trauma. Examination revealed a 1x1 cm firm, tender lump over the lateral aspect of the right nipple. The lesion was completely excised and sent for histopathological examination. The excised specimen showed a globular skin-covered mass measuring 0.7cm in diameter. The cut section was grey white and homogenous with areas of whorling. The overlying skin flap was unremarkable.

**Figure 1:**
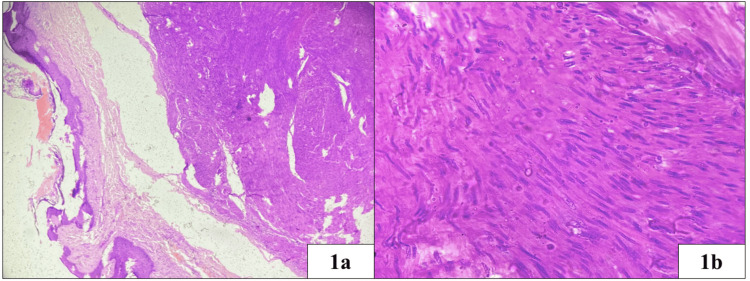
The dermis shows a well-circumscribed mass composed of interlacing fascicles of spindle cells with abundant eosinophilic cytoplasm and oval nuclei with blunt ends. H&E (1a: 100X and 1b: 400X)

**Figure 2:**
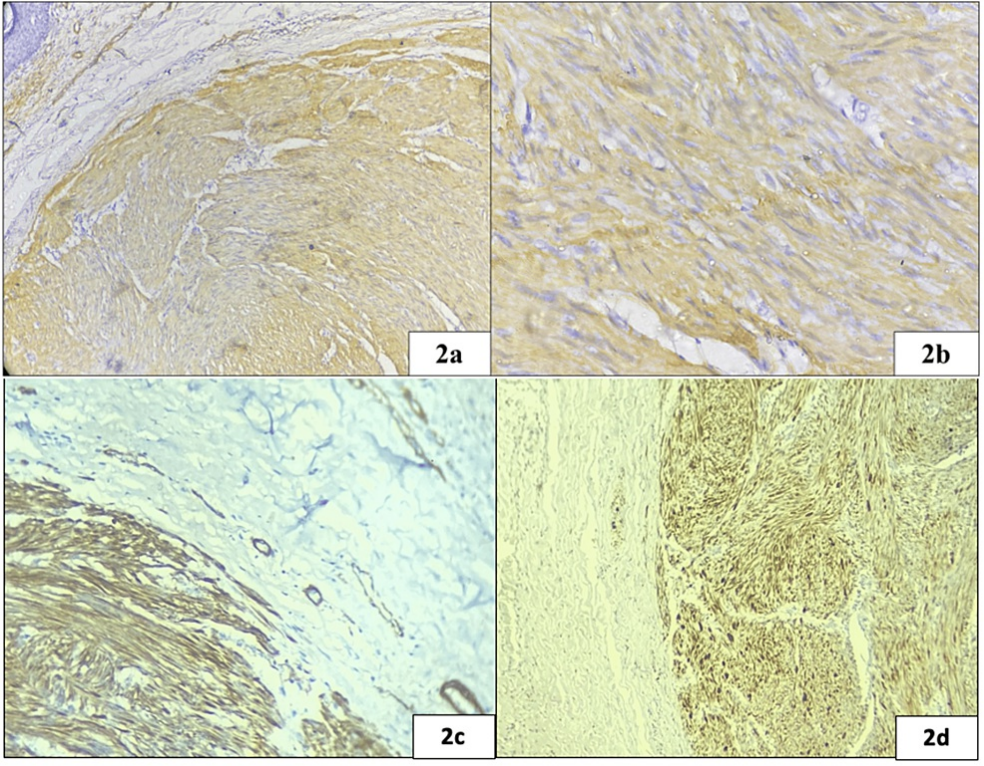
The tumor cells show diffuse cytoplasmic positivity with smooth muscle actin (a: 200X and b: 400X). The tumor cells show diffuse cytoplasmic positivity with caldesmon (2c: 400X). The tumor cells show diffuse nuclear positivity with progesterone receptor (2d: 400X)

Microscopic examination revealed a well-circumscribed, unencapsulated tumor in the dermis composed of spindle-shaped cells arranged in interlacing fascicles and whorls (Figure 1a). The cells had moderate eosinophilic cytoplasm, oval nuclei with blunt ends giving a classical cigar-shaped appearance and inconspicuous nucleoli. No mitosis, atypia or necrosis was seen. The overlying epidermis was unremarkable (Figure 1a, 1b). The cells showed diffuse immunohistochemical expression with smooth muscle actin (SMA) (Figure 2a, 2b) and caldesmon (Figure 2c). A final diagnosis of nipple leiomyoma was made. Tumor cells were also positive for progesterone receptor (PR) (Figure 2d), desmin. Tumor cells were negative for S100. Ki67 labelling index was less than 1%. At a follow-up period of nine months, the patient remained free of recurrence.

## Discussion

Leiomyoma of the nipple belongs to the broader group of genital (cutaneous) leiomyomas. More commonly seen in middle-aged females with a sex ratio of 3:1, the causation has been linked with estrogen and progesterone [2]. Oral contraceptive pills (OCPs) and past history of trauma are other predisposing factors [3]. Nipple leiomyoma has also been reported to occur in males [4], sporadically or associated with gynecomastia. Unlike other cutaneous leiomyomas, nipple leiomyomas are associated with pain less frequently and are generally less than 2 cm in diameter. The present case had a painful lesion. The pain has been attributed to calcium-dependent contraction of the smooth muscle cells [5]. Nipple discharge or retraction is usually not associated. Histopathological examination is necessary and confirmatory for diagnosis. The lesion is usually well-circumscribed and composed of spindle cells with elongated nuclei with blunt ends and eosinophilic cytoplasm. Perinuclear cytoplasmic vacuolization is also common. Other prominent features include stromal fibrosis and varying amounts of interlacing collagen. Immunohistochemical confirmation with SMA and desmin is diagnostic. Sometimes tumor cells are also positive for progesterone receptor (PR) also seen in the present case indicative of hormonal association [4]. The various differential diagnoses that need to be considered are adenoleiomyoma, angioleiomyoma, fibromatosis, myoid hamartoma, myoepithelioma, benign fibrous histiocytoma, fibroadenoma with prominent smooth muscle and leiomyosarcoma [1,4]. Adenoleiomyomas and fibroadenomas can easily be differentiated from leiomyomas by the presence of ductal elements along with smooth muscle proliferation. Angioleiomyomas have prominent slit-like veins with muscular walls intermixed with smooth muscle fibres. Fibromatosis, benign fibrous histiocytoma and myoepithelioma have other components admixed with smooth muscle bundles like fibroblasts, myofibroblasts, and myoepithelial cells. Myoid hamartoma is composed of scattered glandular elements showing apocrine change and benign hyperplasia with smooth muscle differentiation in the stroma. Though atypia and mitosis can be rarely associated with leiomyomas, leiomyosarcomas are more cellular and have increased pleomorphism, mitosis and coagulative necrosis.

Medical treatment to relieve pain can be considered and includes calcium channel blockers and alpha-adrenergic blockers [1]. Definitive treatment includes complete excision with negative margins. Literature is lacking when it comes to data on recurrence. Hammer et al. reported six cases with none showing recurrence in a maximum follow-up period of 12 years [2]. There have been no reports of malignant transformation.

## Conclusions

Nipple leiomyoma is a rare benign lesion at a rare location and needs to be correctly diagnosed microscopically by a pathologist. Immunohistochemistry is a useful tool for differential diagnosis and confirmation of diagnosis. Pathologists should be aware of this rare diagnosis in rare site for timely diagnosis and management of the patient.
